# Potential Protection Effect of ER Homeostasis of N^6^-(2-Hydroxyethyl)adenosine Isolated from *Cordyceps cicadae* in Nonsteroidal Anti-Inflammatory Drug-Stimulated Human Proximal Tubular Cells

**DOI:** 10.3390/ijms22041577

**Published:** 2021-02-04

**Authors:** Charng-Cherng Chyau, Huei-Lin Wu, Chiung-Chi Peng, Shiau-Huei Huang, Chin-Chu Chen, Cheng-Hsu Chen, Robert Y. Peng

**Affiliations:** 1 Research Institute of Biotechnology, Hungkuang University, Taichung 43302, Taiwan; exsior@sunrise.hk.edu.tw (H.-L.W.); g107c105@ms.hk.edu.tw (S.-H.H.); 2 Graduate Institute of Clinical Medicine, College of Medicine, Taipei Medical University, Taipei 11031, Taiwan; misspeng@tmu.edu.tw; 3 Grape King Biotechnology Center, Chung-Li City 320054, Taiwan; gkbioeng@grapeking.com.tw; 4 Department of Nephrology, Taichung Veterans General Hospital, Taichung 40705, Taiwan; cschen@vghtc.gov.tw

**Keywords:** endoplasmic reticulum (ER), oxidative stress, renal HK–2 cells, diclofenac, meloxicam

## Abstract

Nonsteroidal anti-inflammatory drugs (NSAIDs) belong to a class of universally and commonly used anti-inflammatory analgesics worldwide. A diversity of drawbacks of NSAIDs have been reported including cellular oxidative stress, which in turn triggers the accumulation of unfolded proteins, enhancing endoplasmic reticulum stress, and finally resulting in renal cell damage. *Cordyceps cicadae* (CC) has been used as a traditional medicine for improving renal function via its anti-inflammatory effects. N^6^-(2-hydroxyethyl)adenosine (HEA), a physiologically active compound, has been reported from CC mycelia (CCM) with anti-inflammatory effects. We hypothesize that HEA could protect human proximal tubular cells (HK–2) from NSAID-mediated effects on differential gene expression at the mRNA and protein levels. To verify this, we first isolated HEA from CCM using Sephadex^®^ LH–20 column chromatography. The MTT assay revealed HEA to be nontoxic up to 100 µM toward HK–2 cells. The HK–2 cells were pretreated with HEA (10–20 µM) and then insulted with the NSAIDs diclofenac (DCF, 200 µM) and meloxicam (MXC, 400 µM) for 24 h. HEA (20 µM) effectively prevented ER stress by attenuating ROS production (*p* < 0.001) and gene expression of ATF–6, PERK, IRE1α, CDCFHOP, IL1β, and NFκB within 24 h. Moreover, HEA reversed the increase of GRP78 and CHOP protein expression levels induced by DCF and MXC, and restored the ER homeostasis. These results demonstrated that HEA treatments effectively protect against DCF- and MXC-induced ER stress damage in human proximal tubular cells through regulation of the GRP78/ATF6/PERK/IRE1α/CHOP pathway.

## 1. Introduction

Although NSAIDs have long been considered as safe medication practices in the treatment of inflammatory diseases, NSAIDs have received much special attention in the past few years due to the adverse effects in the inhibition of prostaglandin synthesis [1]. Prostaglandins have a diversity of physiological effects, acting as important mediators of inflammation [2]. NSAIDs reduce prostaglandin production by the inhibition of both COX–1 and COX–2, as a result, eliciting various electrolyte disturbances, acute renal failure and chronic renal effects [2,3,4,5,6].

As one of the critical organelles, endoplasmic reticulum (ER) carries out fundamental biological functions including protein folding, protein modification, lipid synthesis and calcium signaling [7]. Under stressful conditions, the unfolded or misfolded proteins accumulate in ER lumen, which triggers ER stress to initiate pathological changes via different mechanisms including proteasome inhibition, mitochondrial dysfunction, and the alteration of key ER components [7,8].

DCF-induced acute renal kidney through induction genomic DNA fragmentation and necrotic cell death of proximal and distal convoluted tubules [9]. Similarly, MXC toxicity has been found from glomerular stasis-related hypertrophy and focal interstitial nephritis in the kidneys [10]. The individual differences of adverse effects from these NSAIDs have been implicated in a variety of inhibitory effects in the cyclooxygenase (COX) regulatory inflammation reactions. DCF has been recognized as a nonselective NSAID inhibiting both COX–1 and COX–2, while MXC is known as a selective COX–2 inhibitor [2]. Andalib et al. reported that a considerable degree of nephrotoxicity resulted from DCF compared to MXC in rats [11]. However, an increasing amount of evidence suggests that the risk of kidney injury associated with the use of NSAIDs is higher than that of the nonusers [3].

*Cordyceps cicadae* (CC) contains abundant biologically active compounds including polysaccharides [12,13], adenosine [14], N^6^-(2-hydroxyethyl)-adenosine (HEA) [15], and ergosterol peroxide [16]. It has been utilized extensively in traditional Chinese medicine to treat chronic renal diseases, heart palpitations, infantile convulsions, and dizziness [17]. In vivo treatment with CC reduced urine albumin, albumin-to-creatinine ratio, and kidney injury molecule–1 (kim–1), alleviated interstitial fibrosis, and suppressed renal α-smooth muscle actin (α-SMA) expression [18]. CC polysaccharides (CCP) exhibit an inhibitory effect on the formation of malondialdehyde and increase the antioxidative enzymes’ activities [16]. CCP significantly ameliorated diabetic renal dysfunction, and apparently slowed down the progression of renal interstitial fibrosis [19]. Preliminary clinical study has ensured its safety by oral ingestion [20].

HEA is a Ca^2+^ antagonist and an anti-inflammatory agent [15,21,22]. In diabetic rats, HEA significantly increased the renal antioxidant level, and at the same time dose-dependently reduced the levels of blood glucose, creatine, BUN, urinary protein, albumin, and proinflammatory mediators [15]. Recently, pharmacological studies about HEA are accumulating [14,15,23,24,25]. HEA is beneficial to renal interstitial fibrosis by preventing inflammation via TGF-β1/Smad and NF-κB signaling pathway [26]. Controversially, HEA induced ROS production and mitochondrial membrane potential depolarization [27], triggering caspase-dependent apoptosis [27], while adenosine possessed anti-inflammatory and antifibrotic bioactivities [28].

The CCAAT/enhancer-binding proteins (C/EBP) are a family of transcription factors strongly implicated in the control of genes involved in intermediary metabolism. The C/EBP homologous protein (CHOP) is considered a key event for endoplasmic reticulum (ER) stress-mediated [29,30]. Without ER stress, CHOP expression is low; however, abrupt and remarkable expression can be induced in response to ER stress through IRE1-, PERK- and ATF6-dependent transcriptional inductions [31].

Whether HEA can protect the kidneys from ER stress resulting from treatment of DCF and MXC has never been documented. We propose that HEA could be beneficial to the prevention of renal damage via suppressing the CHOP-related ER stress, and this work is conducted to uncover such a mechanism of action, to point out the shortcomings of current NSAIDs therapy and comment the possible alternative treatments.

## 2. Results

### 2.1. Isolation and Characterization of N^6^-(2-Hydroxyethyl)adenosine

**Figure 1:**
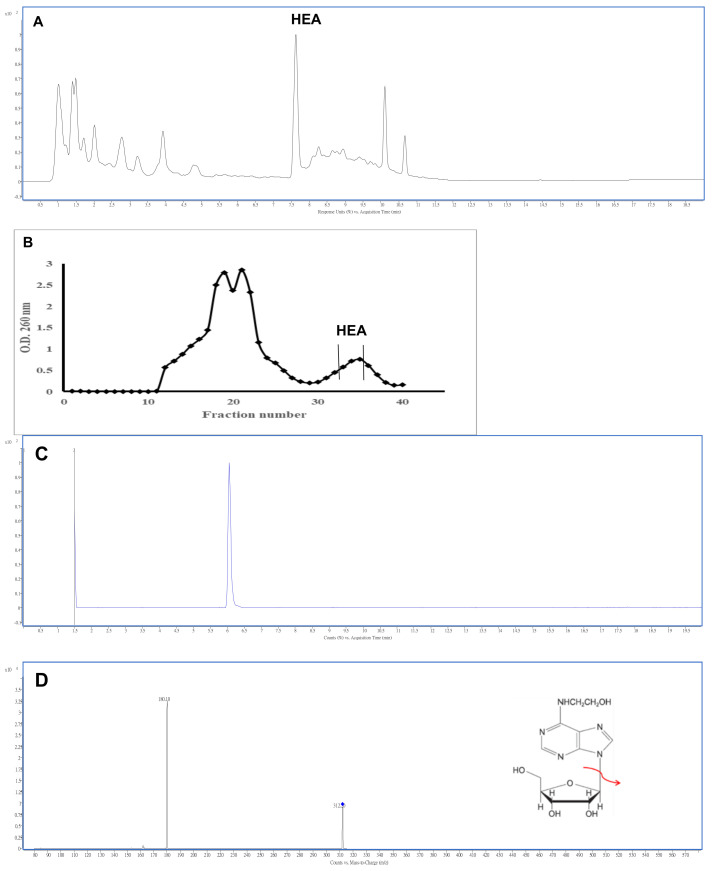
High performance liquid chromatographic analysis of the prepared *C. cicadae* mycelia extract (CCM-2). (**A**) fractionation chromatogram from CCM-2 by gel permeation chromatography Sephadex LH-20. HEA mostly emerged in fractions 33–35. (**B**) total ion chromatogram of HEA (**C**) and the mass spectrum (**D**) of purified N^6^-(2-hydroxyethyl)adenosine (HEA) from *C. cicadae* mycelia extracts; →: *m*/*z* 180 from the MS fragmentation of parent ion 312 in MS/MS analysis.

The freeze-dried powder of CC mycelia when extracted with hot water and precipitated with ethanol, resulted in a yield of 63.15% (*w*/*w*, denoted hereafter as CC-M2) crude extract. After the HPLC analysis, the crude extract prepared from CC mycelia was shown with a major compound of HEA (Figure 1A) existing in the extract, which after separation with Sepharose LH–20 gel column eluted with double distilled water at a flow rate 1 mL/min yielded a fractionation curve as shown in Figure 1B. The main constituent HEA was found mostly merging in fractions 33–35 (Figure 1B), which were then combined, concentrated to obtain the purified HEA product reaching a purity of 95.40% (Figure 1C) in a yield of 1.07 ± 0.04 mg/g dry weight of CC-M2. The total ion chromatography profile of the purified HEA sample was confirmed by HPLC-ESI-(+)-MS/MS analysis in positive ion mode with the protonated pseudomolecular ion [M + H]^+^ at *m*/*z* 312 and the product ion at *m*/*z* 180 (Figure 1C).

### 2.2. The Cell Viability Test-MTT Assay

**Figure 2:**
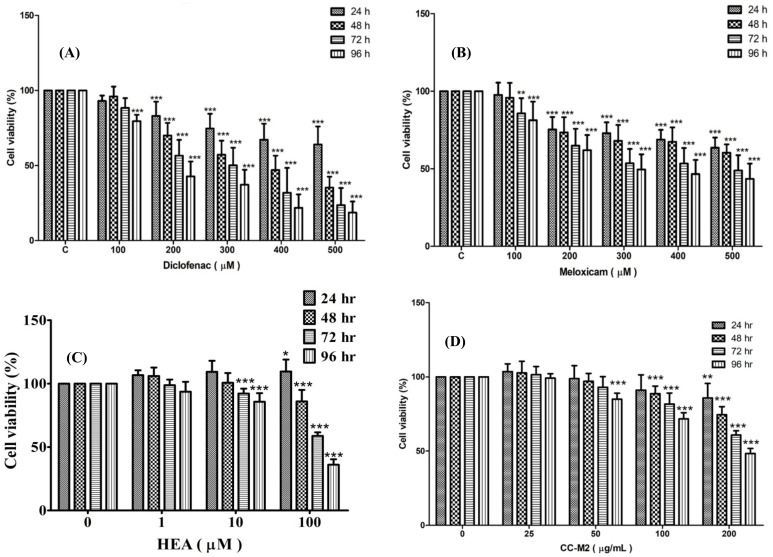
The percent cell viability of HK–2 cells treated with DCF, MXC, HEA, and CC-M2 fraction. HK-cells were treated with DCF (**A**); MXC (**B**); HEA (**C**), and CC-M2 fraction (**D**), respectively. The cell viability was measured at 24, 48, 72 and 96 h, respectively, after treatment. Values are expressed as mean ± SD (*n* = 3). * *p* < 0.05, ** *p* < 0.01 and *** *p* < 0.001 compared with control.

The cell viability as affected by DCF, MXC, HEA, and CC-M2 is shown in Figure 2A–D.

DCF suppressed the cell viability in a dose- and time-dependent manner within the dose range 100 to 500 µM (Figure 2A). The threshold dose to cause the 50% inhibition also depended on the dose and incubation times. As seen, DCF at 100 µM did not cause any inhibition to exceed 50%, even though the samples were incubated for 96 h (Figure 2A). At a dose of 200 µM, 96 h were required to decrease levels to 37% (Figure 2A). At 300 µM, the viability decreased to almost 50% at 72 h. Beyond 400 µM, it took only 48 h to exceed 50% inhibition (Figure 2A).

In contrast, MXC also suppressed the viability of HK-2 cells in dose ranges of 100 to 500 µM (Figure 2B). Beyond 400 µM, the suppression effect was slightly slowed down. To attain 50% inhibition, it took 96 h at 400 µM (Figure 2B).

HEA was almost nontoxic to the HK-2 cells until the dose exceeded 100 µM. At doses within 1–100 µM, the cell viability was suppressed in a time-dependent manner. After being incubated with 100 µM HEA for 48 h the viability was seen to be suppressed to only 10%, and hence it can be considered as “nontoxic” around 10–20 µM of HEA treated for 24 h (Figure 2C). In contrast, the extract CC-M2 was nontoxic to the HK–2 cells up to a dose 100 µg/mL during a 24 h treatment. The 50% inhibition occurred at 96 h after incubation with 200 µg/mL of CC-M2 (Figure 2D).

### 2.3. Production of Reactive Oxygen Species Affected by HEA, DCF, and MXC

**Figure 3:**
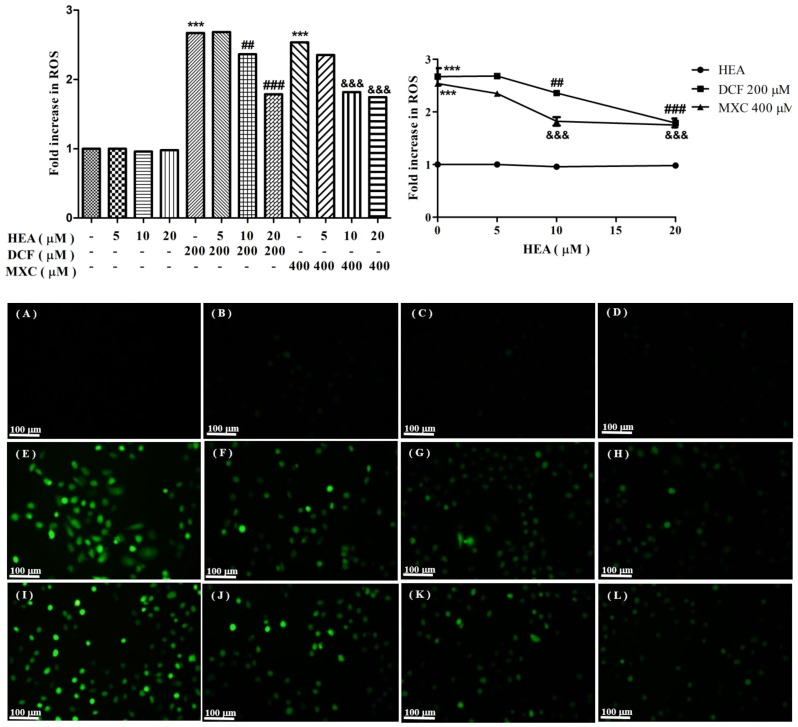
Intracellular ROS induced by DCF and MXC attenuated by HEA. HK–2 cells, pretreated with or without HEA (5, 10 and 20 µM) for 30 min before exposure to DCF (200 µM) and MXC (400 µM) for 4 h, were stained with DCFH-DA and assessed by phase contrast fluorescence microscopy (original magnification, ×100). (**A**): control. (**B**): HEA 5 µM. (**C**): HEA 10 µM. (**D**): HEA 20 µM. (**E**): DCF 200 µM. (**F**): DCF + HEA 5 µM. (**G**): DCF + HEA 10 µM. (**H**): DCF + HEA 20 µM. (**I**): MXC 400 µM. (**J**): MXC + HEA 5 µM. (**K**): MXC + HEA 10 µM. (**L**): MXC + HEA 20 µM. Values are expressed as mean ± SD (*** *p* < 0.001 versus the control group; ^##^ *p* < 0.01 versus the DCF group; ^###^ *p* < 0.001 versus the DCF group; ^&&&^ *p* < 0.001 versus the MXC group).

HEA alone at dose range 5–20 µM was seen to be a nonoxidant (Figure 3B–D) compared to the control (Figure 3A). DCF (200 µM) and MXC (400 µM) stimulated a huge amount of ROS production, which was partially alleviated by HEA in a dose-dependent manner to 1.7-fold of the control HEA (Figure 3E–H for DCF; Figure 3I–L for MXC).

### 2.4. Cell Apoptosis Affected by HEA, DCF and MXC

**Figure 4:**
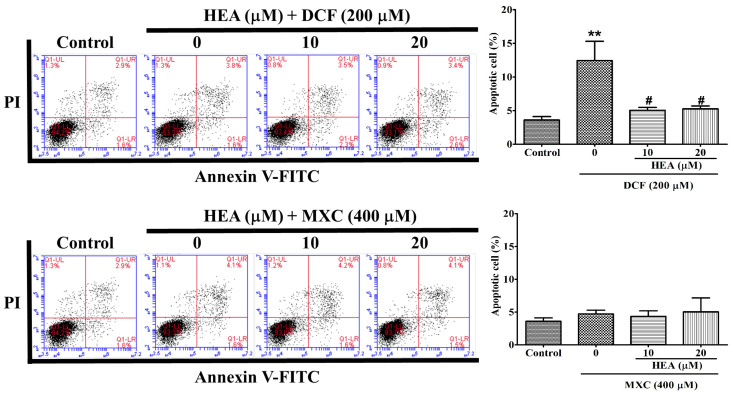
Effects of HEA on the apoptosis of HK–2 cells induced by DCF and MXC. HK–2 cells were pretreated with HEA (10 and 20 µM) for 2 h and then exposed to DCF (top panel) or MXC (bottom panel) for 24 h and subjected to Annexin V-FITC/propidium iodide (PI) double staining assay (left panel). The quantitative analysis of survival and apoptotic cells were performed in triplicate (right panel). Abscissa FL–1: Annexin V-FITC. Ordinate FL–2: PI. Values are expressed as mean ± SD (** *p* < 0.01 versus the control group; ^#^ *p* < 0.05 versus the DCF only group).

The apoptotic assay on the effects of DCF and MXC revealed that DCF but not MXC did induce significant apoptosis of HK–2 cells after treatment for 24 h (Figure 4, left and right panels). The flowcytometric pattern showed apoptosis for the control: death (1.3%), apoptosis (3.6%), and live (95.1%). DCF (200 µM) alone induced death (1.3%), apoptosis (12.4%) and live (86.3%). Combinatorial treatment of DCF with HEA (10 µM) yielded death (0.8%), apoptosis (5.1%), and live (94.1%), while DCF with HEA (20 µM) elicited death (0.9%), apoptosis (5.3%), and live 93.8% (Figure 4, top panel). In contrast, MXC (400 µM) alone did not significantly induce apoptosis (3.6% vs. 4.7%; control vs. MXC). Thus, cotreatment with HEA (10 or 20 µM) did not present significant effects in the cell apoptosis rate (Figure 4, bottom panel).

### 2.5. Effect of HEA on ER-Related Gene Expressions in HK–2 Cells Induced by DCF and MXC

**Figure 5:**
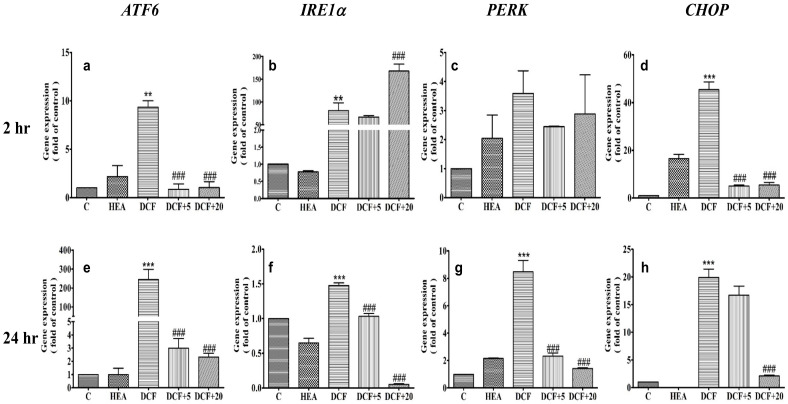
Effect of HEA on gene expression levels in HK–2 cells exposed to DCF. Cells were pretreated with HEA (20 µM) and then exposed to DCF for 2 and 24 h. C: Control. HEA: HEA 20 µM. DCF: DCF 200 µM, DCF + 5: DCF + HEA 5 µM. DCF + 20: DCF + HEA 20 µM). Data are presented as mean ± SD from three independent experiments (** *p* < 0.01 and *** *p* < 0.001 versus the control group, respectively; ^###^ *p* < 0.001 versus the DCF group. Upper panels (**a**–**d**) and lower panels (**e**–**h**) are the expressions of ATF6, IREα1, PERK and CHOP genes exposed to DCF for 2 and 24 h, respectively.

ATF6, IRE1α, PERK and CHOP were the four earliest genes upregulated in HK–2 cells after being treated with DCF 200 µM for 2 h, once upregulated by 9-fold, 98-fold, 4.5-fold and 46-fold were completely downregulated by HEA 20 µM (Figure 5, panels a–d) (*p* < 0.001). At 24 h, AFT6, IRE1α, PERK were stimulated by 250-fold, 1.5-fold and 8.5-fold, respectively, which were ameliorated by HEA down to ≤3-fold, 1.0-fold, and ≤2.5-fold, respectively (Figure 5, panels e–g) (*p* < 0.001). In contrast, at 24 h, the levels of CHOP and IL–1β were upregulated to 20-fold and 400-fold, respectively. While CHOP was suppressed by HEA 20 µM to 17-fold, and to less than 2-fold by HEA 20 µM (*p* < 0.001) (Figure 5, panel h), and IL–1β was attenuated from 400-fold to normal value (*p* < 0.001) (Figure S1).

**Figure 6:**
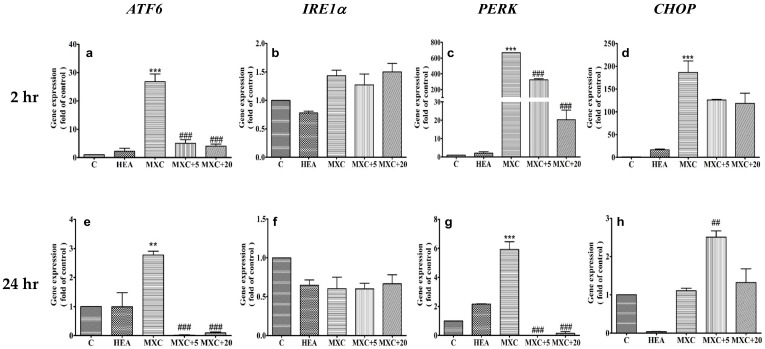
Effect of HEA on gene expression levels in HK–2 cells exposed to MXC. Cells were pretreated with HEA (20 µM) and then exposed to DCF for 2 and 24 h. C: Control. HEA: HEA 20 µM. MXC: 400 µM. MXC +5: MXC 400 µM + HEA 5 µM. MXC+20: MXC 400 µM +HEA 20 µM. Data are presented as mean ± SD from three independent experiments (** *p* < 0.01 and *** *p* < 0.001 versus the control group, respectively; ^##^ *p* < 0.01 and ^###^ *p* < 0.001 versus the MXC group. Upper panels (**a**–**d**) and lower panels (**e**–**h**) are the expressions of ATF6, IREα1, PERK and CHOP genes exposed to 400 µM for 2 and 24 h, respectively.

In contrast, MXC behaved quite differently. At 2 h of treatment, ATF6, PERK, and CHOP were all highly upregulated after being treated with MXC 400 µM, reaching 27-fold (*p* < 0.001), 680-fold, and 180-fold respectively compared to the control (*p* < 0.001) (Figure 6, panels a,c,d). In contrast, IRE1α was attenuated to less than 1.5-fold (Figure 6, panel b) (*p* < 0.001). After 24 h, ATF and PERK were completely attenuated by HEA (Figure 6, panels e,g) (*p* < 0.001). CHOP expression was not completely inhibited until the concentration of HEA reached at 20 µM. Instead, IL–1β once upregulated by 9.3-folds was attenuated to 2.4-fold and normal value after being treated with HEA 5 µM and 20 µM, respectively (Figure S1, panel g) (*p* < 0.001).

### 2.6. Effect of HEA on DCF and MXC Induced the Intracellular GRP78 and CHOP Protein Expession in HK–2 Cell

**Figure 7:**
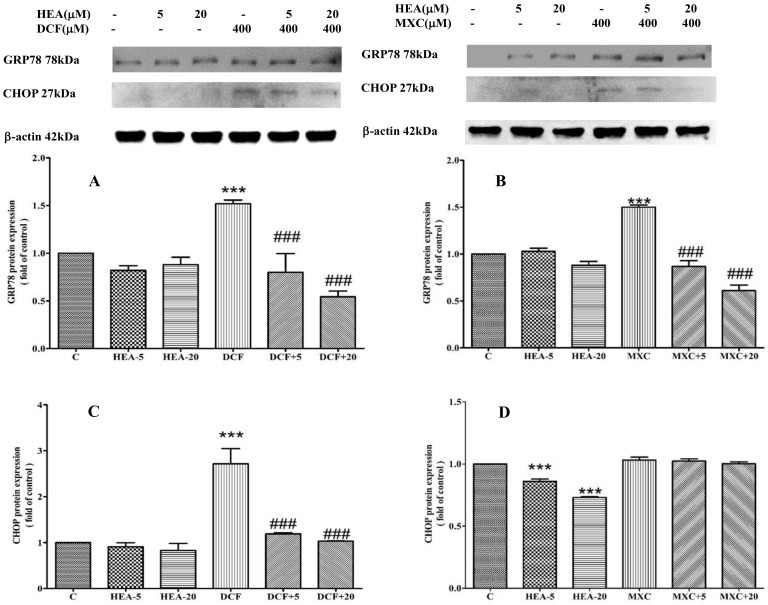
Effect of HEA on intracellular GRP78 and CHOP protein expression levels in HK–2 cells exposed to DCF (**A**,**C**) and MXC (**B**,**D**), respectively, for 24 h. The intracellular protein expression was detected by Western blot. C: Control, HEA: HEA 20 µM, DCF: DCF 200 µM, DCF+5: DCF 200 µM + HEA 5 µM, DCF + 20: DCF 200 µM + HEA 20 µM, MXC: MXC 400 µM, MXC + 5: MXC 400 µM + HEA 5 µM, MXC + 20: MXC 400 µM + HEA 20 µM. Data are presented as mean ± SD from three independent experiments (*** *p* < 0.001 versus the control group; ^###^ *p* < 0.001 versus the DCF or MXC group.

As described above, most of the genes’ expressions, including AFT6, IRE1α, PERK and IL–1β, were upregulated by eliciting MXC 400 µM and DCF 200 µM; only the CHOP gene expression remained the same in the MXC 400 µM treatment at hour 24 (Figure 6 and Figure 7). We investigate whether HEA can influence the MXC and DCF-induced expression of ER-stress markers GRP78 and CHOP in HK–2 cells. We found that the upregulated protein expression of GRP78 by DCF treatment was decreased in HEA treated cells at hour 24 (Figure 7A,C). However, 400 µM MXC did not elevate the protein expression of CHOP as shown in Figure 7D, consistent with the result of gene expression at hour 24 treatment (Figure 6, panel h). In this study, we disclosed that DCF induced a higher ER stress than that MXC did in HK–2 cells.

## 3. Discussion

In this study, the HEA has been isolated by using a Sephadex LH–20 column with a yield of 1.07 ± 0.04 mg/g (on basis of lyophilized *C. cicadae* mycelia extract, CC-M2) and a purity of 95.40% (Figure 1). The limited purity has been found from the coexisting adenosine, an analog of HEA, which is not resolved from the combined fractions under the column chromatography (data not shown). In a similar report, there a content of 1.5 mg/g of HEA was found in *C. cicadae* by using HPLC analysis [20]. Technically, the production yield of HEA can be affected by a variety of fermentation conditions which involve the fungal strain, the dissolved oxygen (DO) level, medium pH, temperature, duration of fermentation, medium ingredients and concentration, and more importantly, the cell density and ratio of inoculum to the medium volume.

Apparently, the cytotoxicity of DCF was higher than MXC (Figure 2A,B), consistent with Ng, et al. [32]. The doses of HEA at 1–100 µM exhibited nontoxic to HK–2 cells at 24 h treatment; however, the longer incubation time (48–96 h), significant inhibition occurred at the dose of 100 µM (*p* < 0.001) (Figure 2C).

Cyclooxygenases (COXs) occur in two forms: the constitutive (COX–1) and the inducible (COX–2) isoforms. COX–1 could provide cytoprotective effects, whereas COX–2 is both inducible and the major isoform for inflammatory responses [2,30]. At low doses, DCF suppresses both COX–1 and COX–2, while MXC at low doses (≤1 μM) is human COX–2 (hCOX–2) selective [2,33]. At higher doses, both DCF and MXC become equivalent inhibitors on both isozymes [34]. As indicated, the doses of DCF and MXC used in the study were 200 and 400 µM (Figure 4), respectively, implicating equipotential inhibition on both isoforms throughout our experiment.

ER stress was associated with some deleterious events including ROS overproduction and apoptosis [8]. We observed that DCFs more significantly induced cell apoptosis in comparison with MXC (Figure 4). Our results demonstrated the findings of Andalib et al., who suggested that DCF presented with a more considerable degree of toxicity than MXC in the kidneys [11].

In the cells, the major source of reactive oxygen species (ROS) is the microsomal monooxygenase (MMO) system, which is composed of cytochrome P450 (P450), NADPH-dependent cytochrome P450 reductase (NPR) and phospholipids [35,36]. Electron uncoupling between NPRs and cytochrome P450 2E1 (P450 2E1) is a major source of ROS on the ER membrane [37].

NSAIDs have been shown to induce reactive oxygen species (ROS) in different cell types including gastric cancer cells [27,38]. DCF stimulated huge production of ROS, inducing oxidative stress and morphological changes in renal tissues consistent with renal damage [1]. An increase in ROS results in increased levels of oxidized proteins, which in turn may alter key intracellular signaling pathways, among which apoptosis plays the key role and causes cell death when significantly activated [38].

Apparently HEA (10–20 µM) alone was a good antioxidant, rather than a prooxidant. Pretreatment with HEA (20 µM) abrogated the ROS production stimulated by these two medicines to only 1.7-fold that of the control HEA (Figure 3), indicating an insufficient dose of HEA. By extrapolation, a dose of 50–80 µM HEA is supposedly required in order to completely attenuate the ROS productions (Figure 3). However, the pretreatment of HK–2 cells with HEA (20 µM) in reality effectively inhibited the upregulation of all important ER-stress related signals (Figure 5 and Figure 6) and readily restored ER homeostasis via scavenging free radicals [39].

When an ER stress occurs, the cell initiates an adaptive response called the unfolded protein response (UPR), the role of which is to maintain protein homeostasis by decreasing the load of unfolded proteins and increasing the protein folding capacity [39,40]. ER stress starts with the activation of three effectors, PKR-like ER kinase (PERK), inositol-requiring enzyme 1 (IRE1), and activating transcription factor 6 (ATF6), following the removal of the chaperone immunoglobulin heavy chain-binding protein (BiP) (GRP78; HSPA5) that maintains them in an inactivated state [8]. UPR also intersects with the integrated stress response that reduces protein synthesis through the inactivation of the initiation factor eIF2α [41].

In response to endoplasmic reticulum (ER) stress, the REDD1 (regulated in development and DNA damage responses) is transcriptionally upregulated through a mechanism involving the activation of PERK, phosphorylation of eIF2α, and increased ATF4 expression [42], promoting CHOP-induced cell apoptosis [7,43,44,45].

CHOP plays an important role in the induction of apoptosis. As mentioned, without ER stress, CHOP expression is low. Abrupt and remarkable expression can be induced in response to ER stress through IRE1-, PERK- and ATF6-dependent transcriptional inductions [31], evidencing rather severe ER stress occurrence when insulted by DCF and MXC (Figure 5 and Figure 6). However, the changes of gene expression in treatment with MXC alone for 2 or 24 h (Figure 6d,h), CHOP gene expression has been restored to its original level of HK–2 cells after the treatment at 24 h, in comparison with the treatment results at 2 h. We suppose that the effects of signaling pathways in the ER stress response were different between the DCF and MXC treatments in HK–2 cells, i.e., with or without the eIF2α-ATF4-CHOP pathway might occur.

The overexpression of CHOP promotes apoptosis in several cell lines, whereas CHOP-deficient cells are resistant to ER stress-induced apoptosis [29,30], pointing to the requirement of a strong CHOP signal (Figure 5 and Figure 6). Otherwise, speculatively, the sustaining period of CHOP also plays an important role in initiating apoptosis, as judged from the results shown in Figure 4, Figure 5 and Figure 6. Furthermore, under certain conditions, ER stress is also able to activate autophagy- and proteasome-dependent proteolysis when misfolded proteins are in excess [39,40].

Taken together the results from Figure 4, Figure 5 and Figure 6, severe ER stress indeed had been initiated by these two NSAIDs (Figure 4 and Figure 6); however, these primary signals were readily attenuated by pretreatment with HEA (10–20 µM) and a restoration of ER homeostasis occurred instead of inducing apoptosis (Figure 4). Such a response can be considered to be an important functions of HEA in abating the effects of oxidative stress and inflammation induced by NSAIDs in cell physiology [15].

The IL–1β gene is involved in the proliferation, differentiation and apoptosis of cells. We showed that the IL–1β gene was upregulated by DCF, while HEA downregulated its expression (Figure S1a,e).

According to a previous report, the upregulation of IL–1β with interleukin–1 receptor-associated kinase–2 (IRAK2) is responsible for triggering ER stress [46,47] and leads to NFκB activation [48], upregulating *i*NOS and stimulating production of NO. Fattori et al. raised the first report demonstrating that DCF induced kidney cell apoptosis, upregulated proinflammatory cytokines, and induced the activation of NF-κB in renal tissue, resulting in acute kidney injury [1]. NFκB is a double-edged sword. Previously, Yin et al. [49] also demonstrated that MXC at a concentration 0.1–1 µM (1 × 10^−7^–1 × 10^−6^ molL^−1^) decreased both endogenous and TNFα-induced NFκB activation [49]. NFκB stimulated inducible nitric oxide synthase (*i*NOS) gene expression to produce NO, which protected hepatocytes from TNF-α- and Fas-mediated apoptosis [50,51]. Combinatorial treatment using NSAID with NFκB inhibitors led to the enhanced induction of apoptosis. We showed that HEA at doses of 20 µM effectively downregulated the expression of NFκB induced by DCF 8 h after treatment (Figure S1b): consistent with this, Lu et al. reported that HEA attenuated proinflammatory responses via suppressing TLR4-mediated NF-κB signaling pathways [23].

To summarize, HEA was beneficial in protecting from renal damages caused by NASIDs, DCF and MXC. Pretreatment with HEA could maintain or restore the ER homeostasis via suppressing ER stress-related elements including ATF6, PERK, IRE–1α, CHOP, ROS, IL–1β, and NFκB.

## 4. Materials and Methods

### 4.1. Chemicals and Antibodies

Antibody against HRP (horseradish peroxidase)-conjugated goat anti(rabbit IgG) antibody (ab97051) was purchased from Abcam (Cambridge, UK). β-Actin (tcba13655), GRP78 (tcea19663) and CHOP (tcba1658) were purchased from Taiclone (Taipei, Taiwan). Diclofenac (DCF) and meloxicam (MXC) were obtained from Novatis (Basel, Switzerland) and Boehringer Ingelheim Pharma GmbH & Co. KG (Biberach, Germany), respectively. MTT and DCFH-DA were purchased from Sigma-Aldrich (St. Louis, MO, USA).

### 4.2. Sources of Cordyceps Cicadae (BCRC MU30106) and HK–2 Cell Line

The fungal strain *C. cicadae* (BCRC MU30106) was gifted by the Grape King Biotech Co. (Taoyuan City, Taiwan). HK–2 (ATCC^®^CRL–2190^TM^) (human kidney–2) cell line, a proximal tubular cell (PTC) line derived from normal kidneys, was purchased from the Biosource Collection and Research Center (BCRC, Hsinchu, Taiwan).

### 4.3. Preparation of the Crude Mycelial Extract

*C. cicadae* (BCRC MU30106) was grown on PDA, transferred to 500 mL flask containing basal medium 200 mL, and incubated at 25 °C with continuous agitation at 120 rpm for three days. The culture was transferred into a 100 L fermenter maintained at 25 °C for four days while continuously stirred at 150 rpm. The fermentation broth was centrifuged and the supernatant was filtered under pressure through a filtering press. The filtrate was lyophilized to obtain the crude mycelial powder [23].

### 4.4. Extraction and Purification of HEA

The mycelial powder was defatted using supercritical extraction carbon dioxide (SCO_2_) under 5000 psi at 60 °C for 1 h, then subjected to solvent extraction with boiling water (1:10 w/v) for 2 h, filtered through 0.22 µm microporous membrane to obtain the aqueous extract which was evaporated under vacuum to one tenth original volume (designated as CCM–1). The filtrate was concentrated under vacuum on a rotary evaporator and freeze-dried (CCM–2). The obtained sample (CCM–2) was then subjected to column separation using the Sephadex^®^ LH–20 column (id × ℓ = 1.5 × 30 cm) and eluted with double distilled water at a flow rate 1 mL/min. The eluent was collected, each fraction from 3 min, until a total of 40 fractions. The OD at 260 nm was used to detect the absorbance of each tube. The fractions from fractions 33–35 were pooled and subjected to further HPLC-ESI-(+)-MS/MS analysis to identify the purified HEA, and then lyophilized (1.07 ± 0.04 mg/g dry weight). The obtained fraction was used for biochemical analyses of HK–2 cells.

### 4.5. Analysis of HEA by HPLC and LC-MS/MS

The chromatographic analysis was carried out on a Hitachi L2130 HPLC system, which was fitted with Phenomenex Hydro-RP 80 Å analysis column (ℓ × id = 150 × 2.1 mm, thickness, 4 µm) and attached to a UV detector (Hitachi L–7455) monitored at 260 nm. The injection volume was 20 µL, the column was maintained at temperature 35 °C, and the flow rate was 0.3 mL/min. The mobile phase was composed of two reagents: phases A and B. Mobile phase A was 10 mM ammonium formate (formic acid, pH adjusted to 6.0). Mobile phase B was acetonitrile containing 0.1% formic acid. The programmed elution condition was: at min 0, A:B = 100:0; at min 3, A:B =100:0. At min 10, A:B = 85:15; at min 15, A:B = 5:95; at min 25, A:B = 5:95; at min 30, A:B =100:0, and at min 50, A:B = 100:0. The compounds having been eluted and separated were further identified with triple quadruple (QQQ) mass spectrometer (Model 6420, Agilent Technologies, Santa Clara, CA, USA) in positive ionization mode with the operating parameters as follows: nitrogen used both as a drying gas at a flow rate of 10 L/min and as a nebulising gas at a pressure of 30 psi, drying gas temperature 325 °C, and a potential of 3500 V applied across the capillary, fragmentor voltage 90 V, and the collision voltage 15 V. Quadrupole 2 was applied for the scanning ions produced by nitrogen collision of ionized compounds in the range 100–800 *m*/*z* at a scan time of 200 ms per cycle. The identification of separated compounds was carried out by comparing their mass spectra provided with UV-Vis spectra.

### 4.6. Cell Culture

HK–2 cells were incubated in a humidified atmosphere with 5% CO_2_ at 37 °C. The culture medium comprised of Dulbecco’s Modified Eagle Medium: Nutrient Mixture F–12 medium (DMEM/F–12) containing 10% of fetal bovine serum and 1% penicillin/streptomycin, which were all purchased from Gibco/Thermo Fisher Scientific, Inc. (Waltham, MA, USA).

### 4.7. Cell Viability Test- MTT Assay

Method of Mosmann et al. [52] was followed. In brief, HK–2 cells at a density 5 × 10^3^ cells/well were seeded onto a 96 well-plate and incubated for 24 h. To each well medium containing different concentration of extracts with DCF (100–500 µM), MXC (100–500 µM), and/or HEA (1–1000 µM) was added as indicated. MTT (final concentration, 0.5 mg/mL) were separately added at hour 24, 48, 72, and 96 and left to stand for 2 h to facilitate the reaction. After the supernatant was sucked off, DMSO was added to dissolve the product formazan crystals. The optical density was read at 570 nm with ELISA Reader (VersaMax, Molecular Devices, Sunnyvale, CA, USA).

### 4.8. Assay for Reactive Oxygen Species

HK–2 cells were seeded onto a 12-well plate at a density of 1 × 10^4^ cells/mL and incubated for 24 h. The cells were pretreated with HEA at dose 5, 10, and 20 µM, respectively, for 30 min. Drugs DCF (200 µM) or MXC (400 µM) was added as indicated and incubated for additional 4 h. The used culture medium was sucked off, replaced with fresh medium containing 10 µM DCFH-DA, and further incubated at 37 °C for 30 min to facilitate the reaction. The medium was sucked off. The cells were rinsed twice with cold PBS and observed under an invert Olympus IX71 microscope.

### 4.9. Analysis for Cell Apoptosis

HK–2 cells at a density 5 × 10^4^ cells/mL were seeded onto a 6 cm petri dish for 24 h. Cells were pretreated with HEA at 10 and 20 µM for 2 h. DCF (200 µM) and/or MXC (400 µM) was added, and the incubation was continued for an additional 24 h. The medium was sucked off and the cells were rinsed with 2 mL PBS. We added 0.05% trypsin 0.02% EDTA to detach the cells, which were collected and centrifuged at 4 °C under 1000× *g* for 5 min. The supernatant was discarded. The sediment cells, rinsed with 3 mL ice cooled PBS, were suspended in 1 mL ice cooled PBS and stained with a mixture containing 100 µL 1× Binding buffer, 5 µL Annexin-V and 5 µL propidium iodide. The cells were dispersed homogeneously, left to stand at 4 °C for 30 min avoiding direct sunlight, and immediately immersed in ice and subjected to flowcytometric analysis using the BD Accuri™ C6 Flow Cytometer (San Jose, CA, USA). The excitation and the emission wavelengths used were λ_ex_ = 488 nm; and λ_em_ = 610 nm, respectively.

### 4.10. Analysis for Gene Expression

#### 4.10.1. Extraction of RNA from Cells

The HK–2 cells at a density 4 × 10^5^ cells/mL were seeded onto a 6 cm dish and incubated for 24 h. Cells were pretreated with HEA at 10 and 20 µM for 2 h, respectively. DCF (200 µM) and MXC (400 µM) were, respectively, added and incubated for 24 h. The used culture medium was sucked off and the cells were rinsed twice with ice-cooled PBS. TRIzol^®^ Reagent (ThermoFisher Scientific, Waltham, MA, USA) (1 mL) was added. The culture was transferred into an Eppendorf vial, 200 µL chloroform was added and vortexed for 10 min, left to stand for 15 min and centrifuged at 13,000× *g* for 15 min. The supernatant (400 µL) was transferred into an Eppendorf vial, 600 µL isopropanol was added, shaken up and down, then left to stand for 30 min, centrifuged at 4 °C under 13,000× *g* for 15 min, and the supernatant was discarded. The sediment was rinsed with 500 µL ethanol (70%), centrifuged at 4 °C under 13,000× *g* for 20 min. The supernatant was discarded. The sediment was redissolved in diethylpyrocarbonate (DEPC) (ThermoFisher) water and stored at −80 °C for use.

#### 4.10.2. Reverse Transcription of RNA to cDNA

The amount of RNA was 1.5 µg as measured with the microspectrophotometer (Clubio, Medclub Scientific, Taoyuan, Taiwan). The operation protocol was carried out by following the instructions given by Takara PrimeScript^TM^ RT Reagent Kit (Takara Bio, Mountain View, CA, USA) (Table S1). The final total volumes for each sample were all adjusted to 10 µL, incubated at 37 °C for 15 min, then reacted at 85 °C for 5 min and stored at −80 °C for use. Supplementary Materials Table S1 depicts the sequence of primers used in this study.

#### 4.10.3. Quantitative Analysis of mRNA Levels

An equal amount of cDNA was used for the subsequent qPCR performed with the SYBR^®^ FAST (KAPA biosystems). The 20 μL reaction mixture contained 9.2 μL of cDNA, 0.4 μL of 10 μM forward and reverse primers, 10 μL of KAPA SYBR FAST qPCR Master Mix (2×). Amplification was performed in an StepOnePlus™ Real-Time PCR System (Applied Biosystems, Foster City, CA, USA). The DNA fragments were amplified for 40 cycles (enzyme activation: 20 sec at 95 °C hold; denaturation: 3 s at 95 °C; annealing: 40 s at 60 °C). The expression of β-actin was determined as the internal control. Relative expression level was calculated using the 2^−∆∆*C*t^ method.

### 4.11. Western Blotting

Following our previous report [53], the expression of proteins, including GRP78 and CHOP in HK–2 cells was measured. In brief, cells were harvested and lysed in 120 µL RIPA and 1 µL protease inhibitor cocktail. The homogenate was centrifuged at 27,210× *g* for 5 min. The supernatant was separated and the protein content was determined and frozen at −30 °C until use. An aliquot of the supernatant, containing 20–30 µg of protein, was measured and mixed with 1/5 × Laemmli sample buffer. The protein samples were then separated on a 10% SDS-PAGE and electroblotted to the nitrocellulose membranes. After blocking with TBS buffer containing 5% nonfat milk, the membrane was incubated overnight at 4 °C with various specific antibodies including GRP78 (1:1000, #TCEA 19663, Taiclone Biotech, Taipei, Taiwan), CHOP (1:1000, #TCEA 1648, Taiclone Biotech, Taipei, Taiwan) and β-actin (1:3000; #MAB1501; Millipore, Billerica, MA, USA), followed by treatment with horseradish peroxidase-conjugated antimouse IgG. The results were visualized with the ECL chemiluminescent detection kit (PerkinElmer, Waltham, MA, USA) and quantified by with the Image J gel analysis software.

### 4.12. Statistical Analysis

Data acquired in this study were presented in a mean ± SD manner and further analyzed by soft GraphPad Prism Program (GraphPad, San Diego, CA, USA). One way analysis of variance (ANOVA) was used for analysis of variations in each group. Tukey’s post hoc test was used for analysis of the significance of difference among the means. A confidence level *p* < 0.05 was considered to be statistically significant.

## 5. Conclusions

**Figure 8:**
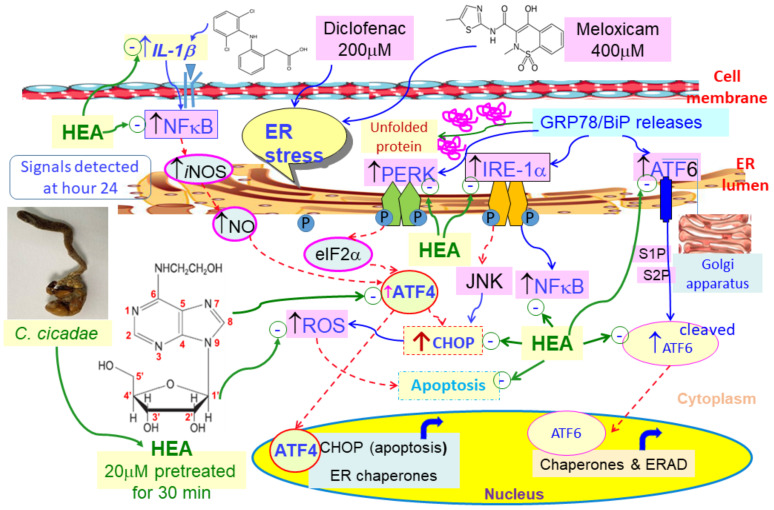
HEA alleviated the nonsteroidal anti-inflammatory drugs (NSAIDs) DCF- and MXC-induced ER stress via the GRP78/ATF6/PERK/ IRE1α/CHOP pathway. The ER homeostasis was disturbed by NSAIDs, which triggered the dissociation of transcription factor 6 (ATF6), protein kinase R-like ER kinase (PERK), and inositol-requiring enzyme 1α (IRE1α) from GRP78/binding protein (BIP). HEA effectively prevented ER stress by attenuating ROS production and gene expression of ATF–6, PERK, IRE1α and CHOP. Solid blue arrows →: signals induced by applied NSAIDs. Solid green arrows →: attenuation by HEA, N^6^-(2-hydroxyethyl)adenosine. Dotted red arrows - - - →: literature-cited pathways.

In this report, an efficient, simple, and cost-effective Sephadex^®^ LH–20 column chromatography was successfully applied to purify HEA from CC mycelia crude extract for the first time. HEA has been revealed with promising protective capability against NSAIDs-induced ER stress in kidney cells. The pathway, as shown in Figure 8, indicated that HEA potentially protected HK–2 cells against ER stress and restored ER homeostasis when exposed to DCF and MXC was via attenuating the early expression of AFT6, IRE1α, PERK, CHOP, ROS, IL–1β, and NFκB when insulted by NSAIDs. Further studies in vivo are required to validate the therapeutic potential of HEA to protect the kidneys from NSAID-induced damage.

## Supplementary Materials

Supplementary materials are available online at https://www.mdpi.com/1422-0067/22/4/1577/s1.

## Abbreviations

ATF6	activating transcription factor 6
BUN	blood urea nitrogen
CCM	*Cordyceps cicadae* mycelium
CHOP	C/EBP-homologous protein
COX	cyclooxygenase
DCF	diclofenac
DCFH-DA	2′,7′-dichlorodihydrofluorescein diacetate
eIF2α	eukaryotic translation initiation factor 2α
ER	endoplasmic reticulum
GRP78	78-kDa glucose-regulated protein
HCC	hepatocellular carcinoma
HEA	N^6^-(2-hydroxyethyl)adenosine
IRE1α	Inositol-requiring transmembrane kinase/endoribonuclease 1α
MTT	3-dimethylthiazol–2,5-diphenyltetrazolium bromide
MXC	meloxicam
NSAIDs	nonsteroidal antiinflammatory drugs
PERK	protein kinase RNA-like endoplasmic reticulum kinase
RIPA	Radio Immuno Precipitation Assay buffer
ROS	reactive oxygen species
