# Supplementary material for: Potential Protection Effect of ER Homeostasis of N6-(2-Hydroxyethyl)adenosine Isolated from Cordyceps cicadae in Nonsteroidal Anti-Inflammatory Drug-Stimulated Human Proximal Tubular Cells
Source: Int J Mol Sci. 2021 Feb 4;22(4):1577. doi: 10.3390/ijms22041577 (PMC7913954; doi:10.3390/ijms22041577)
Supplement: Supplementary file 1 [file ijms-22-01577-s001.pdf]

Supplementary

Potential Protection Effect of ER Homeostasis of N^6-(2-hydroxyethyl)adenosine Isolated from *Cordyceps cicadae* in Nonsteroidal Anti-Inflammatory Drug-Stimulated Human Proximal Tubular Cells

Charng-Cherng Chyau [1,*], Huei-Lin Wu [1], Chiung-Chi Peng [2], Shiau-Huei Huang [1], Chin-Chu Chen [3], Cheng-Hsu Chen [4] and Robert Y. Peng [1,*]

1 Research Institute of Biotechnology, Hungkuang University, Taichung 43302, Taiwan; exsior@sunrise.hk.edu.tw (H.-L.W.); g107c105@ms.hk.edu.tw (S.-H.H.); ypeng@sunrise.hk.edu.tw (R.Y.P.)
2 Graduate Institute of Clinical Medicine, College of Medicine, Taipei Medical University, Taipei 11031, Taiwan; misspeng@tmu.edu.tw
3 Grape King Biotechnology Center, Chung-Li City 320054, Taiwan; gkbioeng@grapeking.com.tw
4 Department of Nephrology, Taichung Veterans General Hospital, Taichung 40705, Taiwan; cschen@vghtc.gov.tw
* Correspondence: ccchyau@hk.edu.tw (C.C.C.); ypeng@sunrise.hk.edu.tw (R.Y.P.); Tel.: +886−26318652; Fax: +886−4−26525386

Table S1. The sequence of primers used in this study.

Name	Sequence (5′ to 3′)
IL-1β -forward	*ccagggacaggatatggagca*
IL-1β - reverse	*ttcaacacgcaggacaggtacag*
IRE1α- forward	*gatcctcagaaacgcccctc*
IRE1α- reverse	*tctgtcgctcacgtcctg*
PERK- forward	*aggacagaggggacagagttg*
PERK- reverse	*taatgaccttttcttccctgctcc*
ATF6- forward	*agtattttgtccgcctgccg*
ATF6- reverse	*gcagaatcccaatcttcatcca*
CHOP(GADD153)- forward	*cagagctggaacctgaggag*
CHOP(GADD153)- reverse	*tggatcagtctggaaaagca*
NFkB-forward	*aacagcagatggcccatacc*
NFkB- reverse	*aacctttgctggtcccacat*

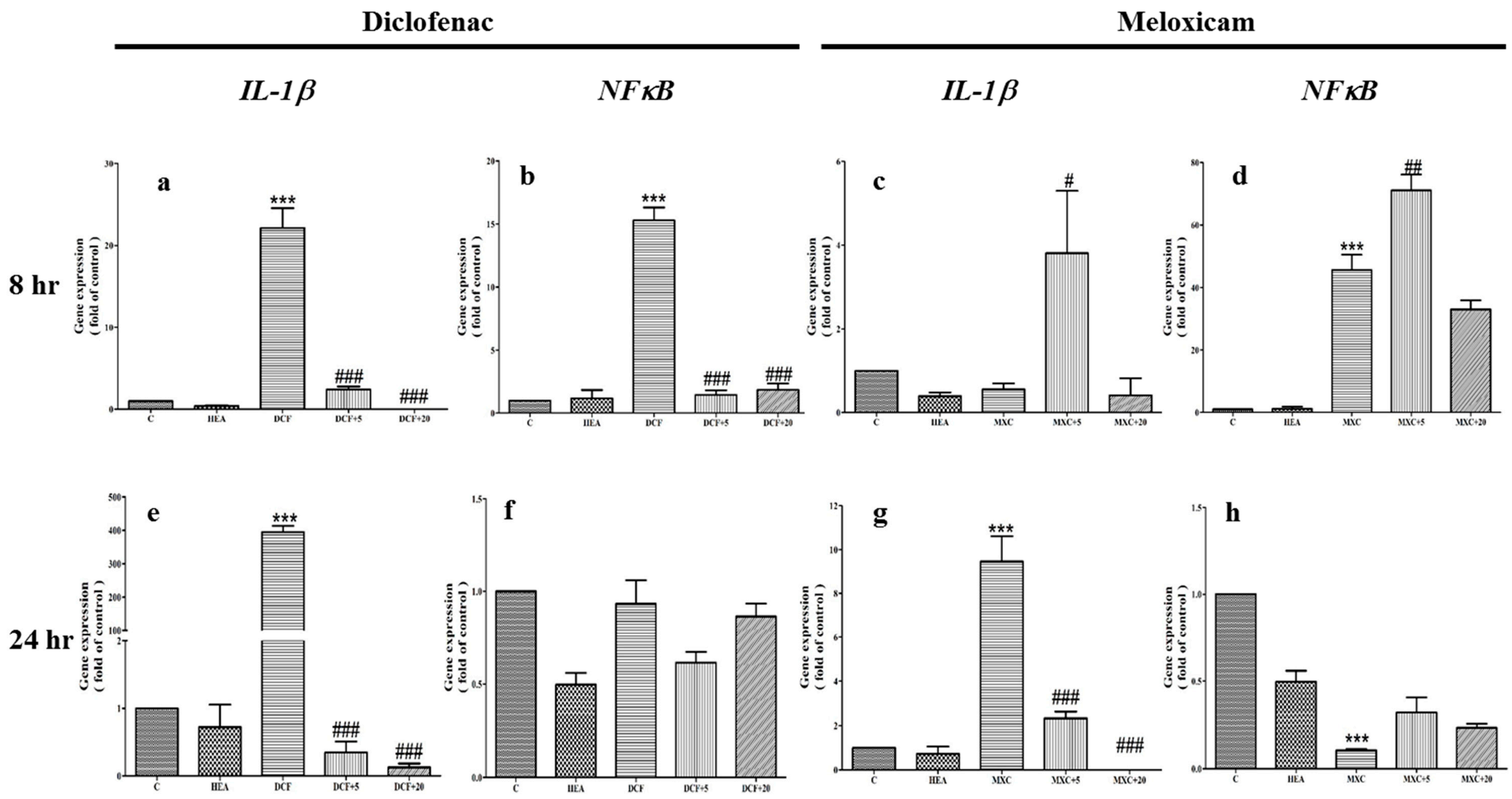

Figure S1. Effect of HEA on time-dependent genes expressions of IL-1β and NFκB in HK-2 cells induced by diclofenac and meloxicam.
C: Control. HEA: HEA 20 μM. DCF: diclofenac 200 μM, DCF+5: DCF+HEA 5μM. DCF+20: DCF+HEA 20μM. MXC: Meloxicam 400 μM. MXC+5: MXC +HEA 5 μM. MXC+20: MXC+HEA 20 μM. Values are expressed as the mean ± SD. ** $P < 0.01$ and *** < 0.001 vs. control group. ### $p < 0.001$ vs. HEA group.
